# Salivary characteristics may be associated with burning mouth syndrome?

**DOI:** 10.4317/jced.58033

**Published:** 2021-06-01

**Authors:** Juan Aitken-Saavedra, Sandra-Beatriz-Chaves Tarquinio, Wellington-Luiz-De Oliveira da Rosa, Ana-Paula-Neutzling Gomes, Adriana-Fernandes da Silva, Matheus-dos Santos Fernandez, Andressa-Goicochea Moreira, Andrea Maturana-Ramirez, Ana-Carolina-Uchoa Vasconcellos

**Affiliations:** 1 Department of Oral Pathology and Medicine, Faculty of Dentistry, University of Chile. Santiago, Chile; 2 Graduate Program in Dentistry, Federal University of Pelotas. Pelotas, Brazil; 3 Diagnostic Center for Oral Diseases, School of Dentistry, Federal University of Pelotas, Brazil; 4 Department of Restorative Dentistry, School of Dentistry, Federal University of Pelotas. Pelotas, Brazil; 5 School of Dentistry, Federal University of Pelotas. Pelotas, Brazil

## Abstract

**Background:**

Burning mouth syndrome (BMS) it is characterized by burning and uncomfortable sensations with no clinical alterations or laboratory findings. The evaluation of the salivary characteristics of people with BMS can help the understanding of the pathogenesis of this condition. This case-control study aimed to trace the salivary profile of women with burning mouth syndrome (BMS).

**Material and Methods:**

40 women with BMS and 40 control women were recruited. Unstimulated salivary flow rate (uSFR), pH, salivary cortisol levels, salivary viscosity, and oral health impact profile (OHIP-14 questioner) were determined. *P*< 0.05 was considered statistically significant.

**Results:**

For uSFR, mean values obtained for BMS and for control group respectively were 0.35 and 0.61 mL/min; for pH, 7.23 and 7.34; for cortisol levels, 0.36 and 0.15 μg/dL; for viscosity values, 31.1 and 45.01 mPas and for OHIP-14 scores, 21.7 and 5.7. To uSFR, cortisol levels, viscosity values and OHIP-14 scores, differences were statistically significant. Salivary cortisol levels and OHIP-14 scores were correlated positively (rho = 0.624; *p*< 0.05).

**Conclusions:**

BMS women have lower uSFR and salivary viscosity and higher salivary cortisol levels that were associated with worse quality of life, compared with the control group.

** Key words:**Xerostomia, Burning mouth syndrome, Viscosity.

## Introduction

Burning mouth syndrome (BMS) is a chronic oral condition that can dramatically undermine the quality of life of affected individuals (mainly adult women); it is characterized by burning and uncomfortable sensations with no clinical alterations or laboratory findings (1). BMS may be a primary process or be attributed to some locals or systemic pathological processes. Although the main cause of primary BMS has not been fully identified, local, systemic, and psychological factors have been associated with its pathogenesis (2). As such, qualitative and quantitative salivary changes that present patients with BMS have been pointed in this direction (3). The evaluation of the salivary characteristics of people with BMS can help the understanding of the pathogenesis of this condition, to estimate the systemic state and even to determine the response to the therapies of the affected persons. Moreover, saliva collection involves simple methods of collecting repeated samples for serial analyses and requires noninvasive methods (4).

Saliva has essential properties to maintain health and homeostasis in the oral cavity (5). Some salivary changes can produce neurological disorders of transduction that can produce alterations in the sensory perception of patients with BMS (5,6). Patients with BMS exhibit significantly more symptoms of depression, anxiety and psychosocial stress levels (6,7) and these characteristics are related to changes in cortisol levels. This hormone is responsible for the regulation of physiological stress and metabolic and immunological functions and could be evaluated in saliva as an indicator of anxiety, stress, and quality of life (7). Although other salivary characteristics, such as the viscosity and quantification of total proteins, may serve as determinants of BMS (8), evidence remain insufficient to establish their association with the etiology of BMS or even if they may be a consequence of the systemic status of BMS in this condition (3).

Although some qualitative and quantitative salivary changes have been evaluated in people with BMS (5-7), findings are still contradictory. This study aimed to trace the salivary profile of women with BMS, compare them with the profile of women without BMS, and associate these results with systemic health, drug use, and effect of BMS symptoms on the quality of life. This study could improve guidance on associated therapies.

## Material and Methods

-Study design and sampling

This was a case-control study conducted in Brazil, between August 2016 and March 2019. The study was approved by the Research Ethics Committee of the School of Dentistry of Federal University of Pelotas (UFPel), Brazil (#2.078.409). Individuals who agreed to participate in the study signed a free informed consent form. The study was performed under the Declaration of Helsinki. A total of 40 women with BMS and 40 control women, referred to the Diagnostic Center for Oral Diseases (DCOD) of the Federal University of Pelotas, from 2016 to 2019 were recruited.

-Determination of the severity of BMS and xerostomia

The International Classification of Headache Disorders criteria were applied to establish the diagnosis of primary BMS: 1) moderate-to-severe, daily, and bilateral burning sensation in the oral mucosa; 2) burning sensation with a duration of at least 4–6 months; 3) burning sensation could remain constant or increase the intensity during the day; 4) burning sensation could improve with food or liquid intake or interfere with sleep; and 5) absence of local and systemic factors that justify burning sensation (2). A questionnaire was used to determine xerostomia (8). The intensity of the symptoms was estimated using a visual analog scale wherein 1 represented the absence of symptoms, and 10 the maximum symptomatic perception experienced by the patient.

A medical history, including information related to current systemic diseases, ongoing medications and smoking and alcohol habits was obtained for all patients. The Eleventh Revision of the International Classiﬁcation of Diseases (ICD-11) was used as criteria to define alcohol habits (9). All volunteers were evaluated by a dentist specializing in oral pathology. Patients also underwent laboratory tests (complete blood cell counts and glycated hemoglobin). Exclusion criteria for cases and control group included the presence of any of the following: previous history of head and neck malignancy, a history of radiation therapy in the head and neck area, chronic thyroid disease, known Sjogren’s disease, any alteration in blood cell counts, those who had prior surgery of the salivary glands, rheumatoid arthritis, contact allergies, pregnant women, history of herpes zoster, with signs and symptoms of buccal lichen planus and patients treated symptomatically or that showed relief after the use of corticosteroids or antifungals about the burning sensation. In the case of patients with type 2 diabetes mellitus (DM2), only those with glycated hemoglobin (hemoglobin A1C) of <7%, which is considered adequate glycemic control, were recruited in the study (10).

-Determination of unstimulated salivary flow rate (uSFR)

Saliva was collected under resting conditions in a quiet room between 9:00 A.M. and 12:00 P.M. The patients were asked to avoid smoking, brush their teeth, or consume food 1h before saliva collection. After 5 min of relaxation, they were instructed to collect their saliva for 5 min and dispense it in preweighed and labeled centrifuge tubes, following the protocol described by Navazesh (11). During the procedure, the participants were instructed to remain seated, stay quiet, and avoid speaking. After 5 min, the tubes with saliva were kept in a container at 5°C for transport to the laboratory of the Diagnosis Center of Oral Diseases (DCOD) of UFPel. Each tube was later weighed through gravimetry, a specific weight of 1.005 g/mL was assigned to the fluid, and the calculated total volume was expressed in milliliters per minute to determine the uSFR.

-Salivary pH measurement

The pH of the saliva samples from each individual was determined using the saliva from the same tube used for SFR measurement. A digital pH meter (PL-600 EZDO-OMEGA model by ISO-9001 regulation) automatically provided the pH with two decimal ranges (12).

-Salivary viscosity

Salivary samples were analyzed with a dynamic rheology technique by using a HAAKE CaBER-1 extensional rheometer (Thermo-Fisher Scientific, MA, USA). The samples were thawed, vortexed, and loaded between 6 mm-diameter plates set within an initial 2 mm range. All the measurements were performed at 37 °C by using 1 mL of each sample. Viscosity was recorded for 150 s of continuous monitoring. Rheometer plates were cleaned initially with ethanol and subsequently with distilled water; they were then air-dried between the evaluated samples. Three measurements were taken per sample, and the mean of each sample was used for statistical analysis (13).

-Quality of life assessment

To determine the different aspects of oral function and quality of life to BMS suffering, we assessed the study participants who were asked to answer a questionnaire for assessing their oral health impact profile (OHIP-14), validated in Brazil (14), and used it to establish the quality of life based on the sum of their answers to the 14 questions. The answer options with their respective values were as follows: 0= never; 1= rarely; 2= sometimes; 3= repeatedly; 4= always. The higher the score (out of a maximum of 56), the worse the quality of life.

-Determination of salivary cortisol

On the day of the test, the saliva samples were thawed completely, vortexed, and centrifuged at 1500 rpm for 15 min at 4 °C. The supernatants were kept at room temperature and analyzed in duplicate using salivary cortisol enzyme immunoassay Enzyme-Linked ImmunoSorbent Assay (ELISA). Spectrophotometers (MFA), whose measuring capacity was 450 nm, were used to determine absorbance. The test protocol was carried out following the manufacturer’s specifications (Salimetrics®).

-Statistical analysis

Descriptive and quantitative data analysis was performed using the Statistical Package for the Social Sciences for Windows 22.0 (SPSS, Inc., Chicago, IL, USA). A Mann-Whitney test was performed to compare the uSFR, pH, salivary cortisol and viscosity between BMS and control group and the average of the intensity of burning sensation among patients with primary and secondary BMS and according to types of drugs used. Spearman test was conducted to associate the levels of salivary cortisol with the impact profile of oral health. *P* < 0.05 was considered statistically significant. According to a pilot study, a sample size of 80 patients (including cases and controls) was determined to have an 80% power assuming a 5% significance level.

## Results

-Baseline characteristics

BMS group consisted of 40 white female patients (age = 62.7 ± 10.8 years; range = 37–84 years). From these, 20 (50%) reported xerostomia. Sixteen patients (40%) were classified as primary BMS and 24 (60%) as secondary BMS. The average intensity of burning sensation in evaluated by visual analog scale was 7.73 (± 2.15); 8.3 (±2.15) for primary BMS and 7.5 (±2.10) for secondary BMS. Ten patients (25%) had no comorbidity. The most frequent additional comorbidity was depression (n=21/52.5%) followed by arterial hypertension (AH) (n=20/50%) and DM2 (n=6/15%). In terms of antihypertensive drugs, the most used were diuretics (n=15/37.5%), followed by drugs of the angiotensin-renin system (ACEI) or blockers or antagonists of angiotensin II receptors (ARAII) (13/32.5%). Six (15%) patients used both types of medication at the same time. For the treatment of depression, 16 (40%) used benzodiazepines and 12 (30%) selective serotonin reuptake inhibitors (SSRIs). For DM2 treatment, 6 (15%) used metformin. Five 5 (12.5%) did not use drugs. No significant differences were observed when compared average burn intensity between patients with primary and secondary BMS, BMS patients with and without any comorbidities neither when compared to patients with AH or depression that used different types of drugs to the treatment of these diseases (*p* <0.05).

The control group was comprised of 40 white women without BMS (age = 48.5 ± 12.35 years; range = 30–66 years). From these, 5 (12.5%) reported xerostomia. Twenty patients (50%) had no comorbidity. The most frequent additional comorbidity was AH (n=15/37.5%) followed by depression (n=8/20%). Ten patients (25%) received diuretics, nine (22.5%) received ACEI or ARA II and 4 (10%) used both types of medication at the same time. For the treatment of depression, 7 (17.5%) used benzodiazepines and 5 (12.5%) SSRIs. For DM2 treatment, 3 (7.5%) used metformin. Nineteen (47.5%) did not use drugs. The concomitant medical conditions, the most frequent drugs, and habits are summarized in Table 1.

**Table 1:**
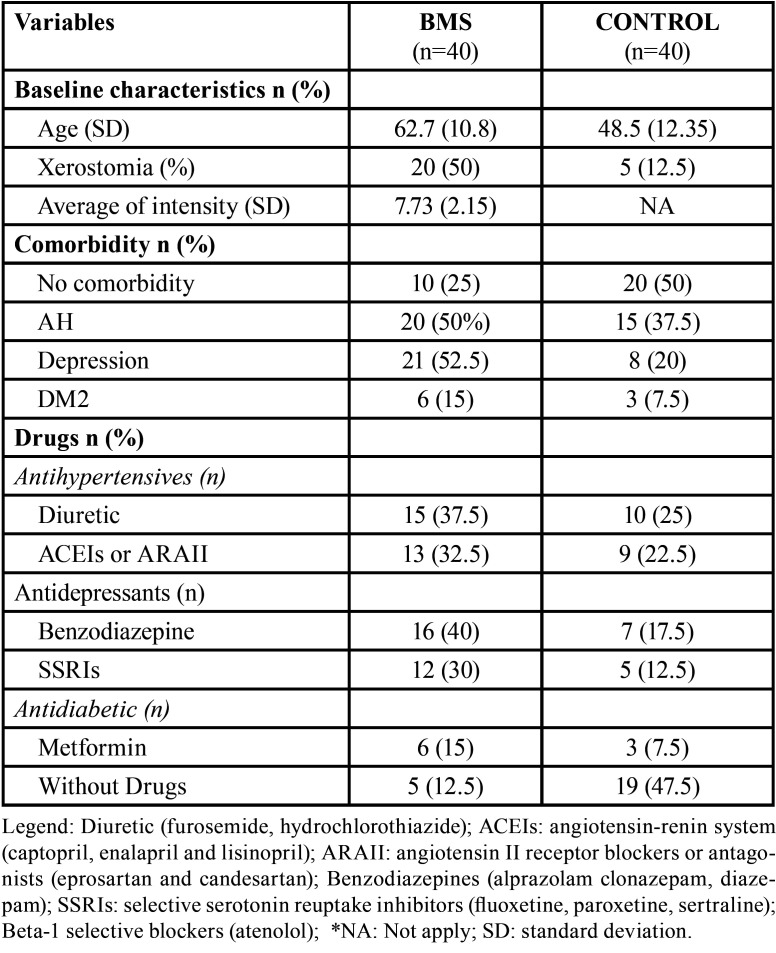
Baseline characteristics of women with and without burning mouth syndrome.

-Salivary characterization

**Figure 1:**
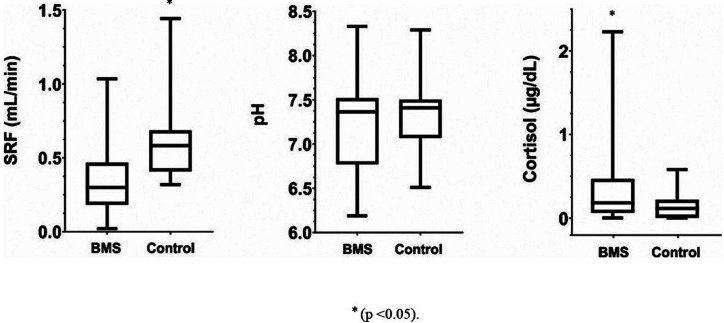
Comparison of unstimulated salivary flow rate (uSFR) and salivary cortisol between women with burning mouth syndrome and women in the control group.

**Figure 2:**
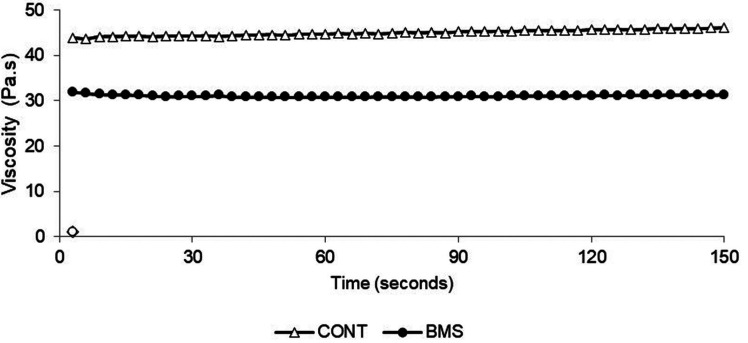
Comparison of the average viscosity of the whole saliva between women with burning mouth syndrome and women in the control group.

The results of salivary characterization are presented in Figures 1 and 2. The mean and standard deviation for pH to BMS and control group respectively were 7.23 (± 0.52) and 7.34 (± 0.49); for uSFR were 0.35 (± 0.24) and 0.61 (± 0.61) mL/min; for cortisol were 0.361 (± 0.47) and 0.152 (± 0.23) μg/dL and for viscosity were 31.13 (± 0.23) and 45.01 (± 0.65) mPas. The BMS group showed higher levels of cortisol and lower values of uSRF and viscosity compared to the control group with statistically significant differences (*p* <0.05). The pH values did not differ between both groups (*p*>0.05).

-Quality of life (OHIP-14 scores)

Regarding quality of life, which was measured with the OHIP-14 questionnaire, women with and without BMS revealed an average of 21.7 (DS 7.27) and 5.7 (DS 4.73), respectively. BMS patients showed worse quality of life with statistically significant differences (*p* = 0.001).

-Correlation between the quality of life (OHIP-14 scores) and salivary cortisol levels

**Figure 3:**
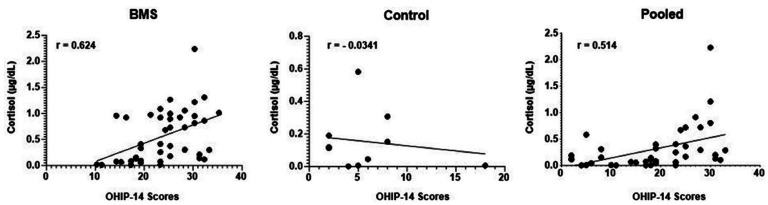
Association between scores ot OHIP-14 (quality of life) and salivary cortisol levels in women with burning mouth syndrome and women in the control group.

Salivary cortisol levels were positively correlated with OHIP-14 scores (r = 0.514 and *P* = 0.0005). When the groups were evaluated separately, we found that salivary cortisol levels were positively correlated with high OHIP-14 scores in the group of women with BMS (r = 0.6242 and *P* = 0.0002) (Fig. 3). No correlation was found between these two variables in the control group.

## Discussion

BMS is an idiopathic condition characterized by chronic pain and a burning sensation in the oral mucosa (1). The prevalence of the syndrome is higher among women, especially after menopause. The mean age of women with BMS observed in our sample agree with the data described in the literature that indicate an average of around 60 years due to biological, sociocultural and psychological factors (1,2,15). The female predominance of BMS increases with age, which may suggest that hormonal changes, especially in the activity of estrogen and progesterone that produce hot flashes, interruption of control mechanisms in menopause, increased night sweating, and emotional lability, play an important role in the etiopathogenesis of the syndrome (16).

Some evidence suggests that the burning symptom may arise from the direct effect of the drugs used in to treat systemic conditions, such as diuretics (5), IECA or ARAII (17) and not necessarily due to the presence of comorbidity. No differences were observed between primary and secondary BMS in terms of the intensity of burning or respect to the presence of xerostomia in the present study. In addition, AH, comorbidity not associated with the diagnosis of secondary BMS, was highly frequent in BMS group (18). Our results agree with the current evidence that does not associate AH with BMS in terms of its etiopathogenesis (15) and the high frequency of this comorbidity observed in BMS group in our study, can be related with a worldwide trend where more than 66% of people over 60 in the world presents HA (19). Another fact that could explain the absence of differences in burning intensity and the presence of xerostomia between primary and secondary is the presence of adequate glycemic values in BMS group (20).

The fact that in our study depression was the common comorbidity in patients with BMS, agrees with previous findings (21). A previous study found that patients with BMS with psychological problems (secondary BMS) have a higher intensity of burning sensation in BMS (2). This phenomenon could be the result of a statistically significant decrease in uSRF as a result of the use of antidepressant drugs, such as benzodiazepines, what would exacerbate the intensity of the burning sensation and could induce xerostomia (2,22). The fact that, in our study, no changes were observed in relation to uSRF could explain the absence of differences for the intensity of the burning sensation and the presence of xerostomia between primary and secondary BMS. In addition, it is indicated that the percentage of BMS patients with xerostomia varies from 10 to 66% (23). This variability and the lack of association between this symptom and BMS in our research, point out that xerostomia in the syndrome may have a multifactorial etiology (5).

Hyposalivation or a decrease in uSRF, can promote the lack of chemical and physical protection of the oral mucosa, facilitating the establishment of BMS (24). According to our results, the uSRF in BMS was statistically lower than that of women without the syndrome. Several factors could be determining this result. The chronic assumption of antihypertensive, anxiolytic and antidepressant medications, on the one hand, and the contextual presence of psychological distress on the other hand, could influence the basal tone of the submandibular, sublingual salivary glands and minor salivary glands, responsible for the non-basal salivary flow unstimulated. These glands are innervated by parasympathetic fibers, which could be affected in BMS in the case of a neuropathic origin of the syndrome. However, an assessment to distinguish the type of salivary secretion would have to be done to confirm this phenomenon (25).

In our study, 25% of women with BMS had a resting salivary flow of less than 0.2% (compared to none in the control group). It is assumed that to have SRF ≤ 0.2 ml/min, it would be necessary that approximately 50% of the glandular parenchyma was affected. In this sense, it has been suggested that glandular hypofunction could be a contributing factor in BMS, a phenomenon that would be more frequent in postmenopausal women (26) and could be enhanced due to the anticholinergic effect of medications used to treat comorbidities associated with aging such as antidepressants. The decrease in salivary flow could influence the reception of stimuli and alter the perception in patients with BMS (5). The association between BMS and the lower salivary flow found in the present study and agreement with the explanation of previous research may be due to the simultaneity of systemic diseases, medication use, aging and even associated glandular damage, which supports the hypothesis of a multifactorial etiology of BMS (27).

According to our research, BMS does not appear to be determined by differences in salivary pH, which has also been described in other research (5). Even if there was not enough evidence found in the literature to establish an association between differences in salivary pH and SMB, it is known that even this salivary characteristic could influence oral sensitivity, it would not necessarily determine the burning sensation (28). The viscosity, related to the energy dissipated during salivary flow (29) should be carefully evaluated about the etiology of BMS (5). In our study, the salivary viscosity of women without the syndrome was significantly higher than that of women with BMS. Even evidence that associates salivary viscosity with the syndrome is scarce, it was described that changes in this salivary characteristic could induce feelings of discomfort associated with BMS (3). Another study showed an increase in salivary viscosity associated with a relief of xerostomia (BMS-associated symptom) in patients treated with capsaicin (13), one possible therapy of the syndrome. Even our results suggest that some salivary characteristics founded in BMS patients could determine suffering of the syndrome, which agrees with previous evidence (5), we must consider that the differences between the frequency of drug consumption and even the difference in age average between our BMS group and the control group, could also influence our results.

Studies have established an association between the poorer quality of life and depression (30). According to our results, women with BMS have a worse quality of life than women who do not present the syndrome possibly related to emotional disorders, such as depression (2,21,22). Evidence has shown that these psychological variables are related to changes in cortisol levels and this hormone can be evaluated in saliva as an indicator of anxiety and stress. Our results are consistent with some studies establishing that patients with BMS have higher salivary cortisol levels compared with those individuals without the syndrome (7). Objective factors such as income, age, weight, and social group, and lifestyle factors such as tobacco and alcohol consumption, drug use, exercise, diet and other aspects associated with quality of life not evaluated in our research, would have to be considered in future studies.

As the pathogenesis of BMS is multifactorial, it is difficult to rule out all the variables that could determine its suffering. Factors such as age, presence of comorbidities (with or without association with BMS), use of drugs used for its treatment, and even some undiagnosed disease, are elements that, even trying to isolate and standardize between in cases and controls groups, is not always possible in their wholeness. Another limitation of the present study was that it did not consider the dose of the drug once it’s described that drug-induced BMS is dose-dependent (17). According to our results, lower USFR, lower salivary viscosity and higher levels of salivary cortisol may be a reflection or part of the etiology of BMS. Our study also revealed that the percentage of depression in BMS women was higher than that observed in the control group and that most women with a syndrome used at least one medication to treat their comorbidities. These factors could also influence the differences found in the salivary characteristics in this study. Even so, salivary characteristics can help to better understand both the etiopathogenesis and the consequences of this still enigmatic syndrome.
